# Early infant's use of visual feedback in voluntary reaching for a spatial target

**DOI:** 10.3389/fpsyg.2013.00520

**Published:** 2013-08-09

**Authors:** Lívia S. Pogetti, Rosana M. de Souza, Eloísa Tudella, Luis A. Teixeira

**Affiliations:** ^1^Human Motor Systems Laboratory, University of São PauloSão Paulo, Brazil; ^2^Movement Analysis and Research Laboratory, Federal University of São CarlosSão Carlos, Brazil

**Keywords:** visuomotor control, visual feedback, infancy, reaching, motor development

## Abstract

Capacity of using visual feedback by infants at the age of reaching onset has been controversial. In this investigation we assessed movement kinematics in the task of reaching for a toy in 5-month-olds, comparing movements performed with the preferred arm under full vision versus visual occlusion. That comparison was made in consecutive periods of visual occlusion. Analysis of results revealed that visual occlusion led to decreased straightness of arm displacement toward the toy as compared to full vision. Longer periods of occlusion did not augment that effect. These results offer preliminary evidence for use of visual feedback early in infants' reaching development. Reconciliation of previous and current findings is made by proposing a hybrid mode of feedback processing for manual control reweighting the roles of vision and proprioception as a function of availability of environmental information.

## Introduction

On the basis of an early review of experimental data, Bushnell (1985) hypothesized that by the age of onset of voluntary reaching control of hand movements toward a target is guided by visual feedback. Experimental evidence following Bushnell's hypothesis has shown increased use of vision for exploratory movements at 5 months of age (Rochat, 1989), and that vision of an object to be grasped leads to more frequent hand-object contacts, longer movement times and increased numbers of movement units in infants from 5 months of age onward (McCarty and Ashmead, 1999). As evidence of early use of vision for manual control, it has been shown that newborn infants compensate for an external load applied to the visible arm, while loading the non-visible arm led to displacement of its original position (Van Der Meer et al., 1995, 1996). More specific evaluation of use of visual feedback from arm movements in reaching has been made by comparing infant's reaching under full vision and reaching for a glowing object in the dark. In the latter visual condition, vision from the reaching arm was precluded by turning off room lights while the object to be grasped was kept visible. Analysis of 15-month-olds revealed that reaching in the dark induced longer movement times and earlier peak velocity in comparison with full vision (Carrico and Berthier, 2008). Those results suggest that visual afference has a functional role early in motor development and that in an advanced phase of infancy it is used as a source of sensory feedback to guide reaching movements.

Employment of the paradigm of reaching for a glowing object in the dark about the age of reaching onset, however, has failed to corroborate the hypothesis of early processing of visual feedback. Clifton et al. (1993) repeatedly tested infants between 6 and 25 weeks of age in a reaching task by comparing the conditions of full vision versus a glowing object in the dark. Results showed that infants made their first manual contact with the object in both visual conditions around 12 weeks of age. That finding suggests that vision was not needed to orient the infants' reaching hand toward the target to make their initial successful voluntary reaching. More detailed kinematic analysis of reaching movements has also failed to detect differences between visual conditions in ages around reaching onset (Clifton et al., 1994; Robin et al., 1996; Babinsky et al., 2012). In a recent investigation, Babinsky et al. (2012) compared infants' reaching for a static target under different visual conditions: full vision, object glowing in the dark, and complete darkness including visual occlusion of the object to be grasped. Results revealed that infants reached faster and decelerated their movements for a shorter period of time in the condition of complete darkness as compared to full vision. Reaching under full vision, though, did not lead to kinematic differences in the comparison with reaching for the glowing object in darkness. Equivalent lack of effect of visual feedback from arm movements early in infancy had been previously observed in reaching movements for static (Clifton et al., 1994) and moving (Robin et al., 1996) targets. From results employing the reaching-in-the-dark technique, then, it seems that by the age of reaching onset visual feedback is not functional in infants' reaching movements.

Taken together, findings reviewed thus far suggest that visual feedback plays a functional role later on in infancy (Carrico and Berthier, 2008), whereas by the age of reaching onset it is not effectively used to guide the hand toward a target to be grasped (e.g., Babinsky et al., 2012). Those findings have been interpreted as evidence for use of proprioceptive instead of visual feedback early in reaching development (Clifton et al., 1993; Robin et al., 1996; Babinsky et al., 2012). A factor limiting conclusions from the paradigm of reaching for a glowing object in the dark, however, is that darkness prevents vision not only from the reaching arm but also from the surround of the aimed object. One could argue, thus, that it is not pure visual feedback that has been manipulated but also potentially relevant environmental information, being a confounding factor in the interpretation of results. Another aspect which might affect reaching performance in the darkness is that precluding vision of the whole surrounding might induce a distinct mode of feedback processing. In the darkness infants might control their movements in a feedforward mode or prioritize proprioception as a source of feedback. From this reasoning, evaluation in the darkness in previous investigations may have inhibited use of visual feedback in reaching movements. In the present study we evaluated the role of visual feedback at the age immediately following reaching onset by occluding visibility of the reaching arm, whereas ambient visual information was maintained available by keeping the lights turned on. Additionally, evaluation was made in consecutive periods of time, testing for a potential cumulative effect of time of visual occlusion. We hypothesized that as time of occlusion increases—and then possible calibration of proprioception by vision diminishes—lack of visual feedback becomes more critical for reaching, leading to poor movement control in comparison with reaching under full vision. A further point of originality in the method used here to investigate infants' processing of visual feedback was assessment of movements performed with the preferred arm only. As intermanual performance asymmetry emerges early following reaching onset (Morange and Bloch, 1996; Morange-Majoux et al., 2000; Rönnqvist and Domellöf, 2006), the hand used for reaching might affect also movement kinematics.

## Materials and methods

### Participants

Thirty infants, 18 females, gestational age range of 37–42 weeks (*M* = 39 weeks, *SD* = 1.3), participated in the study. Inclusion criteria were singleton birth at term, appropriate birth weight, absence of pre- or perinatal complications, normal neonatal neurological records. Exclusion criteria were execution of less than three trials with the preferred arm during the occlusion or post-occlusion experimental phases, or being excessively distressed by the experimental protocol. Eight infants were excluded on the basis of these criteria, while 22 infants were evaluated in all experimental phases. Informed consent for infants' participation was obtained from the respective parents, with approval of experimental procedures by the local university Ethics Committee.

### Task and equipment

**Figure 1:**
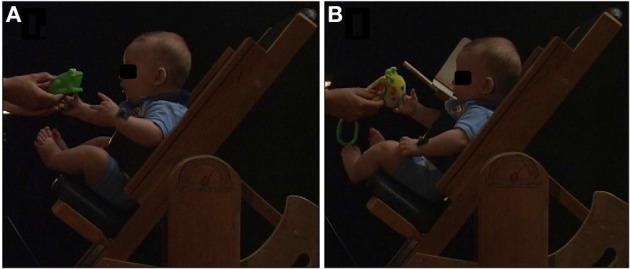
**Representation of the task and experimental setup, showing reaches under full vision (A) and under visual occlusion (B)**.

Infants were positioned in a custom-built infant seat, reclined 50° from the horizontal and positioned at the center of a calibrated area (48 × 32 × 230 cm). The seat allowed for full free range of motion of both arms. Joined to the seat there was an opaque wooden L-shaped screen devised to prevent visual contact with the preferred arm up to the position at which the hand was near the toy. Both screen flaps were 12 × 12 cm, and they were attached near the infant's head to occlude vision of lateral (vertical flip) and under head (horizontal flip) arm movements. The occluding screen did not physically constrain reaching movements toward the toy, and its position was adjusted individually on the left or the right side of infant's head (Figure 1).

To elicit reaching movements, we offered soft and light colorful toys, approximately 5 cm diameter, with similar shapes and weights (approximately 20 g) between them. The toys were manually presented at the midline shoulder height position regarding infant's body, being individually adjusted at the infant's arm-length distance. To assess kinematics of reaching, 5-mm reflective markers were attached with double-sided hypoallergenic tape to both wrists, between the styloid process of the ulna and radius. Four 60-Hz digital cameras were used for offline analysis of hand displacement kinematics.

### Design and procedures

Infants were pseudorandomly assigned to one of two groups: occlusion (OC, 5 females, 5 males) or vision (VI, 10 females, 2 males). The experiment was developed through three phases: pre-occlusion full vision, visual occlusion, and post-occlusion full vision. In the pre-occlusion full vision phase, the toy was presented four times to infants with no visual occlusion of their arms. Toys were presented using both hands to prevent inducing imitation of unimanual use, and they were shifted between trials to increase infant's motivation for reaching. In cases in which a toy failed to induce reaching, another toy was presented in the same manner in the following trial. After reaching, infants were allowed to manipulate the grasped toy for a few seconds. The toy was then withdrawn from the infant's visual field and presented again a few seconds later in the ensuing trial.

In the subsequent visual occlusion phase, infants in the OC group had visibility of their preferred arm prevented by means of the opaque screen during the whole phase, while vision of the opposite arm was not restricted. For infants showing undefined manual preference, half of them had the right and half the left arm (assignment on the basis of order of evaluation) visually occluded. The occlusion phase was divided into periods lasting 1 min of visual occlusion plus the ensuing reaching evaluation. During occlusion periods infants did not perform reaching or manipulative movements, but were free to move their arms without any physical constraint. Immediately following each occlusion period infants were presented with toys at the midline position to obtain four probing trials of reaching with the preferred arm. Probing trials were performed under visual occlusion as well. The nonpreferred arm was not physically constrained, and trials performed with that arm were not analyzed. The other procedures to evaluate reaching movements were the same as described for the pre-occlusion phase. On finishing a set of probing trials, the subsequent period of occlusion was promptly initiated. In cases in which infants became distressed by the experimental procedures they were withdrawn from the seat and delivered for parent's care (*n* = 6). In those cases, infants had opportunity to make visual contact with both arms. When the infant became calm again after an event of distress, he/she was repositioned at the seat and the experiment was resumed from the point at which it was interrupted. This was the main experimental group to test the extent to which absence of visual information from the preferred arm affects reaching performance.

The VI group had full vision of both arms during the occlusion phase, maintaining the same visual condition as for the pre-occlusion phase. Evaluations and intervals were approximately the same as for the OC group. The VI group was the control for possible variation over time of reaching kinematics due to infants staying for some time in the experimental setup and being repeatedly evaluated on reaching for the toys. As infants in both groups frequently had a long delay to initiate reaching movements, full time of the occlusion phase lasted effectively 16–20 min. across infants. We aimed at achieving 8 periods for evaluation in this phase, but some infants did not tolerate that period under evaluation. In those cases the occlusion phase was finished before achievement of the eightieth period.

**Figure 2:**
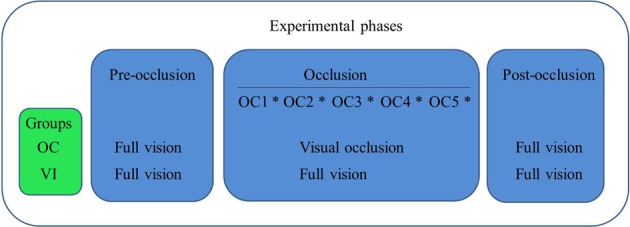
**Schematic representation of the experimental design, showing groups' activities in each phase of the experiment.** In the occlusion phase OC1-OC5 represent each 1-min. period under visual occlusion for OC or full vision for VI. Asterisks between those periods represent evaluation through four reaching trials.

The ensuing full vision post-occlusion phase was initiated immediately after the occlusion phase. In that final experimental phase we adopted the same procedures as described for the pre-occlusion phase, with both groups having full vision of their arms for evaluation of reaching. This last experimental phase had the purpose of evaluating possible after-effects of visual occlusion on reaching kinematics. A schematic representation of the experimental design is presented in Figure 2.

### Analysis

Classification of manual preference was based on frequency of reaches for grasping the toy with the right and the left arm in the pre-occlusion phase. Reaching was classified as unimanual in situations in which a single hand touched/grasped the toy, and bimanual in situations in which both hands touched/grasped the toy following simultaneous displacement of both hands in the direction of the toy for at least one quarter of the trajectory, regardless of uni or bimanual movement onset (based on Fagard, 2000). Analysis was performed on gradients of manual preference, given by the following equation: [(R – L)/(R + L + B)], in which *R* (*L*) is the number of trials executed with the right (left) hand, and B is the number of bimanual reaching movements. Positive (negative) signals indicate more frequent use of the right (left) hand. Infants were classified as right-handers if the gradient was higher than 0.21, as left-handers if the gradient was lower than –0.21, and undefined if gradient was between –0.2 and 0.2.

Three-dimensional reconstruction of reaching movements was carried out through the Dvideow image analyses system (cf. Barros et al., 1999). Raw kinematic data were digitally smoothed using a fourth-order Butterworth filter with cutoff frequency set at 6 Hz. Variables of interest were automatically extracted through Matlab routines after visual inspection of individual trials data. Reaching was assessed on the basis of the following variables: (1) straightness index, calculated as the ratio of the distance between the initial position of the hand and the toy by movement length in the 3D space; (2) number of movement units, calculated as the absolute frequency of peaks sided by valleys in the velocity curve, with differences between upper and lower kinematic landmarks greater than 1 cm/s; and (3) average velocity, calculated as the ratio between the distance traveled by the hand and movement time. Descriptive statistical values are presented for significant differences only.

## Results

### Comparison between right- and left-handers

In a preliminary analysis, it was assessed whether infants showing distinct manual preference had different reaching kinematic profiles. For that analysis, results from the OC and VI groups were pooled together. Fifteen infants were classified as right-handers, 5 as left-handers, and 2 as undefined manual preference. Comparison of right- and left-handers (exclusion of the two cases of undefined manual preference) in the pre-occlusion phase was made through *t* tests for independent measures. Results showed no significant differences: straightness index, *M* = 0.73 (*SD* = 0.14); number of movement units, *M* = 2.54 (*SD* = 1.05); average velocity, *M* = 19.14 cm/s (*SD* = 7.81), *t*-values < 1, *p*-values > 0.3. Following this analysis, data from right- and left handers were treated undistinguishedly in the following analyses.

### Variation of performance between occlusion periods

The minimum number of periods in the occlusion phase tolerated by all infants in both groups was five. For this reason, we evaluated the initial five periods, discarding extra periods achieved by some infants. To evaluate the effect of cumulative visual occlusion on reaching kinematics, the five periods of the occlusion phase were compared separately for each group through a one-way ANOVA for repeated measures. Results from the OC and VI groups indicated absence of significant differences for all dependent variables, *F*-values < 2, *p*-values > 0.1. These results showed that performance was relatively stable over periods of the main experimental phase both under full vision and visual occlusion. Following this analysis, data from the five periods of the occlusion phase were averaged within groups.

### Variation of performance between phases

**Figure 3:**
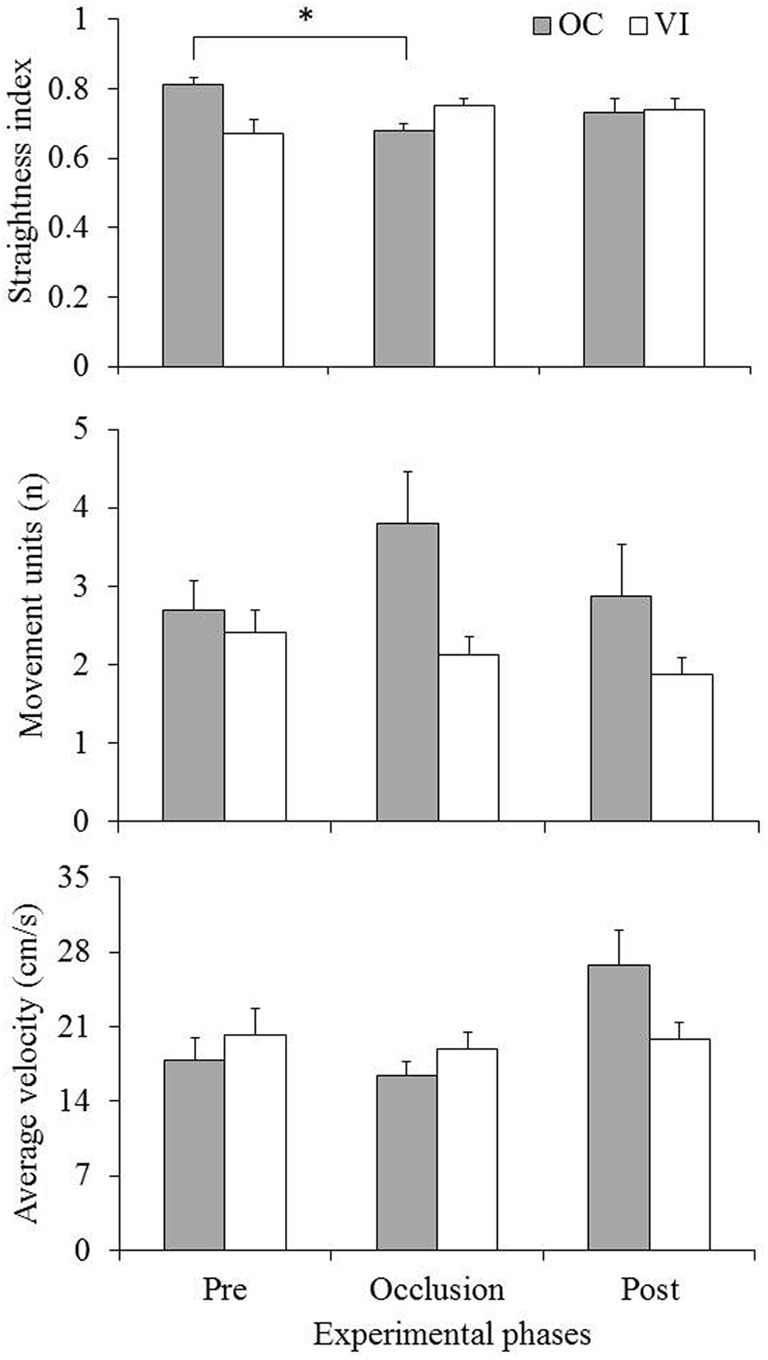
**Mean (standard errors represented by vertical bars) straightness index (upper panel), number of movement units (middle panel), and average velocity (lower panel) for the occlusion (OC) and vision (VI) groups across experimental phases.** Asterisk indicates significant difference (*p* < 0.05).

Mean values (standard errors represented by vertical bars) of the kinematic variables as function of experimental group and phase are presented in Figure 3. Analysis was made through two-way 2 (group) × 3 (phase: pre-occlusion × occlusion × post-occlusion) ANOVAs with repeated measures on the second factor. Analysis of the straightness index (Figure 3, upper panel) indicated a significant group × phase interaction, *F*_(2, 40)_ = 5.45, *p* = 0.001. *Post-hoc* comparisons through Newman–Keuls procedures showed lower values in the occlusion phase in comparison with the pre-occlusion phase for the OC group, while no significant differences were observed in the VI group. Analysis of number of movement units (Figure 3, middle panel) indicated a significant main effect of group, *F*_(1, 20)_ = 4.62, *p* = 0.04. That effect was due to increased values for the OC (*M* = 3.13, *SD* = 1.84) in comparison with the VI (*M* = 2.15, *SD* = 0.84) group. Results from average velocity (Figure 3, lower panel) indicated a significant interaction, *F*_(2, 40)_ = 5.13, *p* = 0.01. *Post-hoc* comparisons indicated increased values in the post-occlusion phase as compared with the other two phases for the OC but not for the VI group.

## Discussion

In this investigation we evaluated use of visual feedback in infants' reaching movements about the age of reaching onset. Evaluation was made by comparing performance under vision occlusion of the preferred arm in successive periods of time and under full vision. We hypothesized that increased periods of visual occlusion of the reaching arm would lead to variation of movement kinematics. Results revealed an effect of visual occlusion in movement straightness, with decline of values in the occlusion phase in comparison with pre-occlusion for the OC group only. For number of movement units the main effect of group suggests an overall difference between groups not associated with visual occlusion, although differences between groups at a descriptive level of analysis tended to be larger in the occlusion phase. Increased average velocity for the OC group in the post-occlusion phase only, on the other hand, cannot be associated with processing of visual feedback. It could be thought to be an effect associated with increased excitement by recovering visibility of the preferred arm instead of being a result associated with movement control. These results represent preliminary evidence for use of vision as a source of movement feedback by the age of reaching onset, although they were inconsistent with the hypothesized effect of increased importance of vision over periods of occlusion.

Previous studies employing the paradigm of reaching for a glowing object in the dark have failed to show an effect of visual feedback in reaching movements by the age of reaching onset (Clifton et al., 1993, 1994; Robin et al., 1996; Babinsky et al., 2012), which is contradictory to the notion that vision predominates for movement control at that age (Bushnell, 1985). Those findings have been interpreted as evidence for use of proprioceptive instead of visual feedback as a predominant source of movement feedback early in reaching development. Use of proprioceptive feedback for controlling the arms might be thought to be adaptive in reaching movements, allowing infants to use vision to monitor spatial position of the target to be grasped while proprioception guides the approaching hand. In this regard, it has been shown that younger infants gaze the target well before reaching initiation and continue to fixate vision on the target following grasping (Sacrey et al., 2012). A point which should be considered on this matter, however, is that for controlling reaching movements from proprioception two frames of sensory information must be integrated: spatial position of the target from vision and dynamic spatial position of the arm from proprioception. To implement that integration the sensorimotor control system of infants at the age of initial voluntary movements should have already developed the capacity to process proprioceptive afference, identifying limb position regarding target position, to make online adjustments during reaching. Evaluation of relative roles of vision and proprioception in the control of manual movements has been made in adults by producing sensory conflicts in a virtual environment (Lateiner and Sainburg, 2003). Results showed that distorted visual information about hand position dominated in movement control over proprioceptive information about the actual hand position. An interpretation for that finding is that the same frame of reference for the target and the hand based on vision bias the control system to use vision instead of proprioception for planning and possibly online regulation of movements. It becomes apparent from those results that intra-sensory integration is prioritized in adults' reaching control by combining vision of the target and of the reaching hand.

Contradictory to findings that by the age of reaching onset infants are unable to use vision as a source of feedback to control manual movements (Clifton et al., 1993, 1994; Robin et al., 1996; Babinsky et al., 2012), our results offer preliminary support for the notion that visual feedback is functional by that age. Observation of reduced straightness during arm visual occlusion suggests that control of arm displacement toward the target was impaired by absence of visual feedback, leading to a more erratic spatial orientation of reaching movements. We did not find the hypothesized cumulative effect of visual occlusion, showing lack of variation of the effect of vision through the successive occlusion periods. From those findings, it may be conceived that under full vision infants are able to process feedback from dynamic spatial location of arm motion through peripheral vision while the object to be grasped is supposedly monitored through focal vision during the hand approach (cf. Sacrey et al., 2012). This interpretation is consistent with results from adults' performance on reaching-and-grasping showing that when peripheral, but not focal, vision is unavailable control of hand displacement is disturbed (Sivak and Mackenzie, 1990). Further on that matter, it has been shown that visual afference from the moving limb in adults' reaching is particularly relevant at the pre-contact movement phase (Churchill et al., 2000). Those analyses on more consistent and well controlled adults' movements suggest that processing of visual feedback is a natural disposition of sensorimotor control in spatially oriented manual actions. In line with that argument, our results showing increased movement straightness under full vision suggest that infants are able to use visual feedback to guide intentional movements in reaching for a spatial target. Another aspect which might be considered on this point is that descriptive analysis of number of movement units suggested that under visual occlusion number of movement corrections was higher. Even though that trend did not reach statistical significance, showing a significant main effect of group only, it is in agreement with the conclusion of use of visual feedback early in infants' reaching.

Reconciliation of previous (Clifton et al., 1993, 1994; Robin et al., 1996; Babinsky et al., 2012) and current findings can be made by the proposition of a hybrid mode of control operating by the age of reaching onset. In the investigation of body balance control in children, it has been found that stance is controlled through a multisensory integration system attributing increased weight to that source of sensory feedback more reliable for the control system (Bair et al., 2007). From that multisensory reweighting model, vision is used when it provides more accurate and reliable sensory information about postural stability. In the opposite case, the role of vision is weakened whereas the role of proprioception is increased (see for recent evidence in adults Jeka et al., 2010; Polastri et al., 2012). A similar flexibility in the use of sensory information can be thought to exist in reaching movements early in infancy. Experimental conditions performed in complete darkness, except by the glowing target to be grasped, may induce use of proprioceptive feedback for movement control. Whereas previous findings in adults indicate that visuomotor control is prioritized under full vision (Lateiner and Sainburg, 2003), results showing lack of effect of vision in infants' reaching (Clifton et al., 1993, 1994; Robin et al., 1996; Babinsky et al., 2012) suggest that proprioception can be used as a reliable sensory source of information about arm displacement toward the target in the ages following reaching onset. Employment of an illuminated environment while reaching for the target, on the other hand, could induce use of the unavailable visual feedback from the moving arm by the infants, leading to decline of some movement control parameters. From this perspective, it is proposed that by seeing the surroundings under full vision during reaching infants' sensorimotor system increased the weight of visual feedback in motor control while the weight of proprioception was diminished. In this case, our experimental setup seems to have made movement control more sensitive to visual occlusion of the reaching arm, offering thus support for the notion of early processing of visual feedback at the age of reaching onset. An experimental test of this proposition could be made in further studies by comparing reaching movements between darkness and illuminated-room arrangements. A limitation in the method used in this study was that, although vision of arm movements were occluded during most of its displacement toward the target, in the short period preceding hand-target contact vision of the reaching hand was available for the infants. As this feature might weaken the effect of visual occlusion, an improvement of the method should be pursued in further investigation using this technique.

### Conflict of interest statement

The authors declare that the research was conducted in the absence of any commercial or financial relationships that could be construed as a potential conflict of interest.
